# Beyond human intentions and emotions

**DOI:** 10.3389/fnhum.2013.00099

**Published:** 2013-03-27

**Authors:** Elsa Juan, Chris Frum, Francesco Bianchi-Demicheli, Yi-Wen Wang, James W. Lewis, Stephanie Cacioppo

**Affiliations:** ^1^Psychology Department, University of GenevaGeneva, Switzerland; ^2^Department of Physiology and Pharmacology, Center for Neuroscience, West Virginia UniversityMorgantown, WV, USA; ^3^Geneva University HospitalGeneva, Switzerland; ^4^Academy of Psychology and Behavior, Tianjin Normal UniversityTianjin, China; ^5^Department of Neurobiology and Anatomy, Center for Neuroscience, West Virginia UniversityMorgantown, WV, USA; ^6^High-Performance Electrical NeuroImaging Laboratory, Department of Psychology, Center for Cognitive and Social Neuroscience, The University of ChicagoChicago, IL, USA

**Keywords:** social neuroscience, embodied cognition, intention understanding, love, neuroimaging, fMRI meta-analysis

## Abstract

Although significant advances have been made in our understanding of the neural basis of action observation and intention understanding in the last few decades by studies demonstrating the involvement of a specific brain network (action observation network; AON), these have been largely based on experimental studies in which people have been considered as strictly isolated entities. However, we, as social species, spend much more of our time performing actions interacting with others. Research shows that a person's position along the continuum of perceived social isolation/bonding to others is associated with a variety of physical and mental health effects. Thus, there is a crucial need to better understand the neural basis of intention understanding performed in interpersonal and emotional contexts. To address this issue, we performed a meta-analysis using of functional magnetic resonance imaging (fMRI) studies over the past decade that examined brain and cortical network processing associated with understanding the intention of others actions vs. those associated with passionate love for others. Both overlapping and distinct cortical and subcortical regions were identified for intention and love, respectively. These findings provide scientists and clinicians with a set of brain regions that can be targeted for future neuroscientific studies on intention understanding, and help develop neurocognitive models of pair-bonding.

## Introduction

Throughout the past three decades, a growing number of studies have shown that one understands actions and intentions of other people by shaping one's understanding and anticipation of the environment based on one's own motor system (Jeannerod, 2001; Van Overwalle and Baetens, 2009; Becchio et al., 2012). Theories on embodied cognition and simulation extend these findings by suggesting that reading the intention of others occurs through a direct and automatic matching process between observed and performed actions, and via the re-activation of the bodily states that were originally active during past self-related sensori-motor experiences—as if the observers were “reliving” the observed motor experiences (Rizzolatti et al., 2001; Rizzolatti and Craighero, 2004; Niedenthal et al., 2005; Lewis et al., 2006; Niedenthal, 2007; Rizzolatti and Sinigaglia, 2008; Grafton, 2009). The recent development in neuroimaging sheds lights on the neural activations mediating this mechanism (Grafton, 2009; Becchio et al., 2012). For instance, neuroimaging studies show that reading intentions of others recruit brain areas that are also activated when someone performs the same action (Grafton, 2009, for review; Ortigue et al., 2009; Rizzolatti and Sinigaglia, 2010). Interestingly, these brain areas can be divided into two functionally separable brain networks (Grafton, 2009; Ortigue et al., 2009). The first, referred to as the “action observation network” (AON), involves an inferior fronto-parietal network (FPN) and includes a subset of areas that are associated within the putative human Mirror Neuron System (hMNS; Grafton et al., 1996; Rizzolatti and Craighero, 2004; Desmurget et al., 2009; Grafton, 2009). This AON system is thought to be particularly important for integrating sensori-motor information during perceptual judgments about actions (Rizzolatti and Craighero, 2004), and also for understanding hand-object interactions and intentions on the basis of embodied cognitive mechanisms (Iacoboni et al., 2005; Rizzolatti and Sinigaglia, 2007; Grafton, 2009). The second brain network, referred to as the “social-network” (SN), recruits brain areas involved in social interaction (Grafton, 2009; Sugiura et al., 2009; Wakusawa et al., 2009; Canessa et al., 2012). This SN includes the medial prefrontal cortex, precuneate cortex, insula, and amygdala (Wheatley et al., 2007; Grafton, 2009). Both networks include the posterior part of the superior temporal sulcus (pSTS); the superior temporal gyrus (STG), the middle temporal gyrus (MTG), and the part of the angular gyrus that is near the ascending limb of STS (Allison et al., 2000; Pelphrey et al., 2004; Thompson et al., 2005, 2007; Pelphrey and Morris, 2006; Materna et al., 2008). Interestingly, the STS region, notably its posterior part (pSTS), is also recruited by relatively low level processes such as observation of visual biological motion (Jellema et al., 2000), auditory biological actions (Bidet-Caulet et al., 2005; Gazzola et al., 2006; Lewis et al., 2011), and other operations such as social inferential processing in tasks requiring mentalizing, and theory of mind (Grezes et al., 2004; Saxe et al., 2004; Schultz et al., 2004; Grossman et al., 2005; Frith and Frith, 2006; Brass et al., 2007; Van Overwalle and Baetens, 2009).

Although these findings provide valuable information about the brain mechanisms involved in the understanding of actions performed by strangers, they do not tell much about the brain mechanisms involved in the understanding of a significant other [a person with whom the participant intends to be with (i.e., a participant's partner in an intimate relationship or a best friend in an companionate relationship)]. To date, studies on intention understanding have been largely based on functional magnetic resonance imaging (fMRI) studies in which participants have been considered as strictly isolated entities i.e., focusing mostly on the action type rather than on the relationship with the agent and the observer. However, people typically spend most of their time in social settings interacting with significant others. Research shows that a person's position along the continuum of perceived social isolation/bonding to others is associated with a variety of physical and mental health effects (Cacioppo and Cacioppo, 2012). For instance, people who subjectively feel isolated live shorter lives than those who feel they have strong, dependable, meaningful social bonds (Cacioppo and Patrick, 2008; Cacioppo and Cacioppo, 2012 for review).

As a consequence, there is a health-related need to better understand the functional dynamic of our brain during actions performed in an interpersonal context. This is critical as we spend much of our lifetime interacting with significant others, acquaintances, as well as strangers.

A growing body of research in psychology highlights the importance of studying the processing of significant others by demonstrating the influence of implicit processing of significant others (compared to strangers) on the individual's perception and cognitive processes. For instance, evidence suggests that the emotional bond between an actor and a perceiver may facilitate mutual intention perception, with a stronger bond associated with faster intention understanding (Cutting and Kozlowski, 1977; Ortigue and Bianchi-Demicheli, 2008; Ortigue et al., 2010a).

Inspired by the theories on embodied cognition and simulation theories, one explanation for this facilitation effect is that intention understanding may be based, in part, upon mechanisms of self-expansion among significant others. Through self-expansion mechanisms, a collective unconscious mental representation may be formed among individuals who share self-characteristics, values, and actions in a common environment (Agnew and Etcheverry, 2006). In line with evolutionary theory's claim that intense emotional experiences during a lifetime (e.g., passionate love) may be a central human motivation to expand one's self (Aron and Aron, 1996; Barkow et al., 1992), the self-expansion theory of pair-bonding is a hallmark in dyadic relationships (Aron and Aron, 1996). As an illustration, couples in love often refer to one another as the “better half” or the “completion of oneself,” and they refer to “We” rather than “I” (Hatfield and Sprecher, 1986; Hatfield and Rapson, 1993; Aron and Aron, 1996), therefore suggesting that there is a cognitive expansion of their self in a beloved, and vice versa an integration of the beloved's values and characteristics in their self (Hatfield and Walster, 1978; Aron and Aron, 1996; Ortigue and Bianchi-Demicheli, 2008). From a cognitive and social viewpoint, self-expansion means that each partner makes a decision (conscious or not) to include the significant other in their own mental self-representation (Aron and Aron, 1996), allowing the formation of a shared mental representation of the self and partner. Recent neuroimaging studies of love (Cacioppo et al., 2012, for review) provide further support in favor of this self-expansion model of love by demonstrating a recruitment of self-related brain network in people who are in love (see Cacioppo et al., 2012, for review). Based on these recent findings in social neuroscience and relationship science (Ortigue et al., 2009, 2010a,b; Cacioppo et al., 2012; Canessa et al., 2012) and based on the self-expansion theory of pair-bonding (Aron and Aron, 1996; Bianchi-Demicheli et al., 2006; Ortigue and Bianchi-Demicheli, 2008; Ortigue et al., 2010a,b), we hypothesized a common pattern (notably within SN) of activation between love and intention tasks (Ortigue and Bianchi-Demicheli, 2008). The rationale for identifying areas of overlap between tasks that involve love and intention is that love varies as a function of the extent to which an individual prefers or desires interaction with another person. This preference or desire, in turn, may activate networks associated more with behavioral intentions in everyday life.

To test this hypothesis, we statistically explored the neural similarities and differences of the neural bases between intention and passionate love for a partner by performing a meta-analysis of fMRI studies involving intention understanding and love, respectively.

## Materials and methods

### Literature search

We performed a systematic review of functional neuroimaging studies of intention understanding and passionate love, respectively. All papers and books in the literature published up to May 2011 (inclusive) were considered for this review, subject to two general limitations: the publication had to be a manuscript, chapter or book, and the title and abstract had to be available in English. Materials were identified through computer-based search, as described below.

### Selection criteria for intention understanding literature

Our systematic computer-based search was based on the published literature of functional neuroimaging studies on intention understanding using MEDLINE library through PubMed database. Key words used for this search were “intention understanding,” “action understanding,” “fMRI,” and “neuroimaging.” Publications were selected on the basis of the following criteria: (1) fMRI neuroimaging studies; (2) with healthy adult participants, and (3) paradigms included stories (text or cartoons) or video-clips on intention understanding only. In all selected studies, participants' instruction was either to observe intentions, to infer the intentions of the actions performed by others, or to answer questions about the intention of the actions (i.e., “why”). In all the studies we classified as “intention understanding” agents were strangers (unfamiliar people) only. A list of the studies and contrast conditions for intention understanding are shown in Table A1.

### Selection criteria for passionate love literature

We similarly performed a computer-based search of functional neuroimaging studies on passionate love using MEDLINE library through PubMed database. Expanding on our earlier study (Ortigue et al., 2010b), we used the key words “love,” “couple,” “fMRI,” and “neuroimaging.” Publications were selected on the basis of the following criteria: (1) fMRI neuroimaging studies; (2) with healthy adult participants in love with a partner, (3) paradigms included viewing partner's face or partner's name, i.e., tasks related to their beloved partner. A list of the studies and contrast conditions for passionate love are shown in Table A2.

### Meta-analysis methods

**Figure 1:**
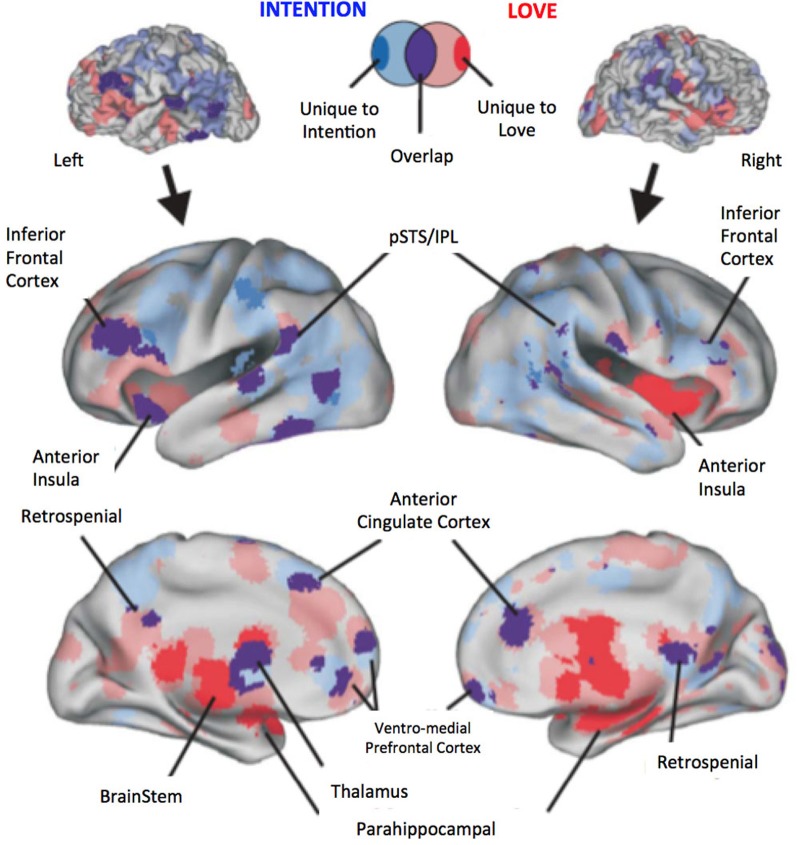
**Meta-analysis results revealing brain regions and networks unique to either Intention Understanding (blue) and Passionate Love (red) and statistically significant overlap between the two (purple).** Upper panel illustrates a typical brain surface model. Lower panel illustrate a slightly inflated rendering of the PALS atlas cortical surface to facilitate visualization of the resulting activation foci. Brain activations significant at *p* < 0.001, FWER corrected. Variations from transparent to solid colors indicate the following terminology: Transparent red, all passionate love study only foci; Transparent blue, all intention study only foci; Purple, overlap of above conditions; Solid red, unique to passionate love only studies; Solid blue, unique to intention only studies.

To provide readers with a synthesized and statistical view of the common and different brain networks mediating intention understanding and passionate love, we analyzed the distribution of peak coordinates related to intention understanding (Figure 1, blue), passionate love (red), and regions common to both (purple). Using techniques reported previously by our group (Lewis, 2006; Ortigue et al., 2010b; Cacioppo et al., 2012), we adopted a Multi-level Kernel Density Analysis (MKDA) approach (Wager et al., 2009). This approach quantitatively tested for consistency and specificity of regional activation across the two sets of studies: it minimizes biases such as having one study that reports many activation foci from dominating the meta-analysis, and it accounts for the smoothness of reported data, false-positive rates, and statistical power. Thus, the reported peak coordinates within a study's contrast maps are weighted in the meta-analysis by study quality and sample size. We calculated the number of statistical contrast maps that activated each voxel in the brain using 10 mm kernel (roughly matching the three dimensional spatial resolution of the reported data). Monte Carlo simulations (10,000 iterations) were used to obtain a threshold and establish statistical significance against a null hypothesis that activated regions in the resulting pair-wise contrast maps are not spatially consistent (i.e., that they are randomly distributed throughout the brain). The use of the distribution of maximum values provides a strong control of family wise error rate and is an established method for multiple comparisons correction (Nichols and Holmes, 2002). All voxels (which constitute the various brain region volumes) whose density exceeded the 99.9th percentile value under the null hypothesis were considered significant (i.e., Family Wise Error Rate corrected for spatial extent at *p* < 0.001). Brain coordinates obtained using the MKDA method above were entered into the SPM8 software program (Wellcome Trust Centre for Neuroimaging, London; http://www.fil.ion.ucl.ac.uk/spm/) using Anatomy Toolbox version 18 (Eickhoff et al., 2005) in order to facilitate identification and labeling of each activation peak. The MNI coordinates and volumes of significantly overlapping clusters (brain foci) were extracted using the AFNI software plug-in 3dcluster (Cox, 1996). Displayed localizations were further validated by visual inspection relative to the Duvernoy and Bourgoin brain atlas (Duvernoy and Bourgoin, 1999). To visualize the meta-analysis results (Figure 1), the significantly overlapping contrast indicator maps for the intention studies and the love studies, and the intention-overlap-love studies were projected (using MNI-Talairach coordinate space) onto the left and right hemisphere Population-Average, Landmark- and Surface-based atlas (PALS atlas), which is an atlas of cortical surfaces that represent the averaged cortical surfaces of 12 individuals (http://brainmap.wustl.edu; Van Essen, 2005). The left and right cortical surfaces were inflated to reveal major sulci of the brain to facilitate viewing of the data.

## Results

Based on our search criteria, we found a total of 25 fMRI studies. This included 17 studies (21 experimental paradigms) for intention understanding (Table A1; Pelphrey et al., 2004; Walter et al., 2004; den Ouden et al., 2005; Iacoboni et al., 2005; Hamilton and Grafton, 2006, 2008; Wang et al., 2006; Brass et al., 2007; Buccino et al., 2007; Ciaramidaro et al., 2007; de Lange et al., 2008; Ortigue et al., 2009; Liew et al., 2010; Newman-Norlund et al., 2010; Ramsey and Hamilton, 2010; Carter et al., 2011; Jastorff et al., 2011) and eight studies (10 experimental paradigms) for passionate love (Table A2; Bartels and Zeki, 2000; Aron et al., 2005; Ortigue et al., 2007; Kim et al., 2009; Zeki and Romaya, 2010; Stoessel et al., 2011; Xu et al., 2011; Acevedo et al., 2012), involving a total of 457 participants. The number of participants included in each study ranged from 10 to 36 (for further details see Tables A1, A2).

Results confirm previous studies by demonstrating that understanding intentions of strangers involved the brain areas involved in both SN and AON, including areas sustaining embodied cognition, simulation, and self-other perception (such as vMPFC, BA6, MTG/STG, Angular gyrus, see Table A3; Figure 1, blue) as well as in the precuneus and the left anterior cingulate cortex, and the right middle and posterior cingulate cortex. In addition, activations in subcortical areas, such as putamen activity were also observed (see Table A3 for further details). Similarly, results on passionate love activations reinforced previous studies (e.g., Acevedo et al., 2012 and Cacioppo et al., 2012) by demonstrating a distributed cortical and subcortical network of SN as well as brain areas known to be involved in partner preference, rewarding experiences, goal-directed actions, habit formation, and decision-making (for further details see Table A4).

### Common brain network between intention understanding and passionate love

Our fMRI meta-analysis revealed a shared brain network between intention understanding and passionate love (Figure 1; purple; Table A5) that includes brain regions sustaining social cognition, embodied cognition, mentalizing about self-other, such as bilateral pSTS/IPL, bilateral inferior frontal cortex (IFC), ventro-medial prefrontal cortex (vMPFC), anterior insula as well as brain regions involved in the mesolimbic and nigrostriatal dopaminergic pathways (caudate nucleus, thalamus, putamen, and parahippocampal area; Table A5).

## Discussion

The present research highlights a shared network between love and intention, which includes (1) areas that overlap with areas related to dopamine circuits; and (2) several regions implicated in social cognition, embodied cognition, attachment, mental state representation, and self-representation. These results are consistent with previous studies indicating that both love and intention involve goal-directed and rewarding behaviors towards a specific partner. The recruitment of dorsal parts of the striatum, such as the caudate and putamen, which are innervated by dopamine coming from both the VTA and substantia nigra, is in line with recent work in animals showing that these brain areas are critical in the development of a pair bond and conditioned partner preference (Pfaus, 1999, 2009; Young et al., 2005, for review), as well as in the activation of stereotyped motor patterns (habits) related to conditioned incentive cues (Everitt and Robbins, 2005). Although dopamine transmission in the ventral striatum has been shown to be stimulated in the presence of both unconditioned and conditioned rewarding incentive cues stimuli (Pfaus et al., 2001; Aragona et al., 2003; Postuma and Dagher, 2006; Pfaus, 2009, for review), responses made to conditioned rewards appear to involve more dorsal striatal networks, among which are outputs of the dorsal striatum to cortical regions such as the insula (Postuma and Dagher, 2006)—a brain region which binds integrated visceral feedback with emotional and cognitive responses (Craig, 2002; Ibanez et al., 2010; Berntson et al., 2011; Cacioppo et al., 2012).

The overlap between passionate love and intention understanding in brain areas, such as the vMPFC, is consistent with a growing body of studies unraveling the recruitment of this brain area during tasks that require introspections about self and by tasks that require inferences about the minds of others perceived to be similar to self (Jenkins et al., 2008). The activation of the anterior insula is also consistent with earlier studies from our laboratory and others implicating a role in meta-representations of self (for further details see, Cacioppo et al., 2012). Other overlapping areas were also activated, such as left and right IFC and MTG/pSTS with a slight left hemisphere lateralization. These areas are in close proximity to classical Broca's and Wernicke's language regions, which entail frontal and temporo-parietal regions that are more highly interconnected via the arcuate fasciculus white matter fiber tracts in humans relative to chimpanzees and to monkeys (Rilling, 2008). One intriguing prospect is that the evolution of these interconnected inferior fronto-parietal regions may relate to pair-bonding and degree of social attachments, as well as other more uniquely human qualities such as language (Kim et al., 2010).

By identifying specific functional brain regions and networks in a large sample of healthy subjects, the present analysis reinforces the consistency and specificity of the brain regions that are being reported in the burgeoning body of studies on love and intention understanding. Interestingly, by revealing an additive brain network for both intention understanding and passionate love, the present findings offer a new way to look at the neurobiology of the loving mind during embodied cognition through the lens of a specific subset of AON and SN regions of interest. This provides scientists and clinicians with a unique and strong rationale to further investigate neurocognitive models that may explain the modulations of these common regions and brain networks in future studies on intention understanding in dyads.

One limitation of the present meta-analysis study was some of the fMRI studies of intention contrasted the condition of interest either to resting state(s) (e.g., Iacoboni et al., 2005; Wang et al., 2006; Newman-Norlund et al., 2010), while others used a variety of different control conditions. Consequently, the resulting activation patterns (Figure 1) must be interpreted with this caveat in mind. However, the goal of the present study was to provide a first pass glimpse at determining (and identifying) candidate brain regions or networks that may show preferential or selective activation to scenarios related to embodied cognition, pair-bonding, and self-expansion mechanisms. The present results do provide novel support for an overlapping brain network between these mechanisms. Future fMRI studies investigating the processing of significant others (compared to strangers) during intention understanding in the same paradigm is thus warranted/needed. The systematic neuroscientific study of the modulations of the neural network for understanding the intentions of significant others in healthy subjects, neurological patients after brain damage, and patients suffering from chronic social and affective disorders (such as autism), will provide critical insights on the spatio-temporal dynamics of self-expansion and embodied mechanisms that may mediate dyadic interactions, notably among couples.

## Financial disclosure/funding

The authors disclosed receipt of the following financial support for the research and/or authorship of this article: Swiss National Science Foundation (Grant #PP00_1_128599/1 to Stephanie Cacioppo), the Mind Science Foundation (Grant #TSA2010-2 to Stephanie Cacioppo, Francesco Bianchi-Demicheli), NCRR NIH COBRE Grant E15524 (Grant #E15524 to the Sensory Neuroscience Research Center of West Virginia University to James W. Lewis), the MOE Project of Key Research Institute of Humanities and Social Sciences (Grant #12JJD190004 to Yi-Wen Wang), and the Program for New Century Excellent Talents in Universities (Grant #NCET-11-1065 to Yi-Wen Wang).

### Conflict of interest statement

The authors declare that the research was conducted in the absence of any commercial or financial relationships that could be construed as a potential conflict of interest.

## Appendix

**Table A1:** **List of fMRI studies on intention understanding**.

**Study (first author listed)**	**Year**	**No of subjects**	**No of women**	**No of right-handed**	**Stimuli**	**Experimental comparisons**
Brass	2007	15	7	15	Video-clips	Implausible > ordinary motion
Buccino	2007	20	10	20	Video-clips	Attend to intention > viewing
					Video-clips	Incorrect unintended motion > ordinary motion
Carter	2011	17	8	15	Video-clips	Human reaching movements: goal shift > goal miss
Ciaramidaro	2007	12	6	12	Comic strips	Intentional (communicative) > physical event
					Comic strips	Intentional (social) > physical event
					Comic strips	Intentional (private) > physical event
de Lange	2008	19	10	19	Pictures	Ordinary > extraordinary intentions
den Ouden	2005	11	11	Not specified	Scenarios	Intentional action > physical event
Hamilton	2006	20	11	19	Video-clips	Repetition suppression for action goal
Hamilton	2008	20	13	20	Video-clips	Repetition suppression for action outcome
Iacoboni	2005	23	15	23	Video-clips	Hand grasping > rest
Jastorff	2011	15	9	15	Video-clips	Non-rational > rational movements
Liew	2010	18	8	18	Video-clips	Hand gestures > still control
Newman-Norlund	2010	18	10	18	Video-clips	Object-directed actions > rest
Ortigue	2009	24	0	24	Video-clips	Repetition suppression for intention
Pelphrey	2004	12	7	12	Video-clips	Incorrect > correct motion
Ramsey	2010	25	17	24	Video-clips	Repetition suppression for object-goal
Walter (1)	2004	13	7	13	Comic strips	Intentional action > physical event
Walter (2)	2004	12	6	12	Comic strips	Intentional action > physical event
Wang	2006	12	6	12	Scenarios	Attend to face > rest
Total		306	161	291		

**Table A2:** **List of fMRI studies on passionate love**.

**Study (first author listed)**	**Year**	**No of subjects**	**No of women**	**No of right-handed**	**Stimuli**	**Experimental comparisons**
Acevedo	2012	17	10	17	Faces	Partner > high familiar acquaintance
					Faces	Partner > close friend
Aron	2005	17	10	17	Faces	Beloved > familiar neutral acquaintance
Bartels	2000	17	11	16	Faces	Beloved > friend
Kim	2009	10	5	10	Faces	Beloved > friend (early)
					Faces	Beloved > friend (late)
Ortigue	2007	36	36	36	Names	Beloved > friend or stranger
Stoessel	2011	12	6	12	Pictures	Beloved > erotic (happy condition)
Xu	2011	18	10	18	Faces	Beloved > familiar neutral acquaintance
Zeki	2010	24	Not specified	Not specified	Faces	Beloved > neutral
Total		151	88	126		

**Table A3:** **Intention understanding activation peaks**.

**Label**	**Left hemisphere**				**Right hemisphere**			
	**MNI coordinates**			**Number of voxels**	**MNI coordinates**			**Number of voxels**
	***x***	***y***	***z***		***x***	***y***	***z***	
vmPFC					6	52	−12	4000
Inferior frontal gyrus (p. Opercularis) (BA 44: 20%)					46	10	27	17904
Superior frontal gyrus (BA 6: 50%)	−18	2	67	7048				
SMA (BA 6: 20%)	−6	20	51	6648				
BA 6 (20%)					20	−5	48	40
Precentral gyrus (BA 6: 60%)					36	−12	58	4120
Precentral gyrus (BA 44: 30%)	−46	7	33	29,424				
BA 45 (10%)					37	33	0	3184
Middle temporal gyrus/STG	−47	−50	18	73,992				
Heschls gyrus (Insula/Ig1: 60%; TE1.1: 50%; OP2: 30%)	−33	−28	13	464				
Gyrus ambiens					46	−6	−23	7688
Angular gyrus (IPC/PFm: 20%; PGa: 20%; hIP1: 10%)					47	−46	24	73,608
Precuneus (SPL/7P: 20%; SPL/7A: 20%)	−2	−55	47	17,744				
Anterior cingulate cortex	−3	49	11	10,424				
					7	41	29	7928
Middle cingulate cortex (BA 6: 10%)					10	−6	44	2864
Posterior cingulate cortex					12	−41	10	7736
Putamen	−26	10	−6	4120				
Fornix	−4	−2	10	4120				
Calcarine gyrus (BA 17: 50%; BA 18: 10%)					16	−82	14	4088
Calcarine gyrus (BA 17: 70%; BA 18: 20%)					23	−99	−2	3424
Middle occipital gyrus (IPC/PGp: 30%)	−35	−84	29	3024				
Optic radiations	−26	−34	10	2336				

**Table A4:** **Passionate love activations peaks**.

**Label**	**Left hemisphere**				**Right hemisphere**			
	**MNI coordinates**			**Number of voxels**	**MNI coordinates**			**Number of voxels**
	***x***	***y***	***z***		***x***	***y***	***z***	
vmPFC					10	39	−11	2744
	−5	49	−8	9384				
dlPFC	−7	51	22	5056				
SMA (BA 6: 60%; BA 4: 10%)					10	−18	61	24,760
Postcentral gyrus (BA 3b: 60%; BA 1: 10%; BA 6: 10%)					61	−3	24	6784
Superior temporal gyrus (TE3: 10%)					63	−14	−6	3656
Superior temporal gyrus (IPC/PFcm: 10%; OP1: 10%; TE1.1: 10%)	−56	−28	8	4120				
Posterior middle temporal gyrus (V5: 10%)	−55	−67	2	3312				
Inferior temporal gyrus	−40	−8	−28	4120				
Middle cingulate cortex					10	5	32	16
Supramarginal gyrus (IPC/PFm: 70%; IPC/PF: 30%; IPC/PGa: 20%)					62	−45	25	7040
Supramarginal gyrus (IPC/PF: 60%; IPC/PFm: 40%; IPC/PFcm: 10%)	−63	−46	25	4096				
Superior parietal lobule (SPL/7A: 40%; BA2: 30%; SPL/5L: 30%)	−23	−50	53	3128				
PGa (10%)					40	−48	15	16
Calcarine gyrus (BA 17: 100%; BA 18: 30%)					9	−90	1	22,440
Cuneus					18	−70	24	6192
Thalamus	−3	−12	1					284,552
Cerebellum					42	−57	−28	7512
					34	−45	−48	6656
	−36	−78	−40	3896				
**PASSIONATE LOVE ACTIVATIONS (USING SPHERICAL KERNEL OF 3 mm)**								
Superior frontal gyrus	−24	12	66	648				
Posterior superior temporal gyrus (IPC: 50%)	−50	−38	18	600				
Superior medial frontal gyrus	−9	27	59	1288				
	−10	52	16	632				
Middle orbital gyrus/orbitofrontal					2	62	−8	648
Gyrus rectus (orbitofrontal)	−4	60	−24	648				
Middle frontal gyrus	−30	54	2	648				
Superior anterior medial frontal gyrus					8	54	22	1272
					11	65	5	1264
Inferior frontal gyrus (p. orbitalis)					35	33	−8	1200
Inferior frontal gyrus (p. triangularis)	−37	28	0	16				
	−37	26	−2	16				
	−33	35	−2	824				
					56	28	8	648
Middle frontal gyrus					30	47	4	1200
	−30	44	11	344				
Anterior cingulate cortex					8	40	9	1152
					8	28	16	648
	−6	42	−6	600				
Middle cingulate cortex					4	−22	42	648
					12	−22	34	616
					14	−20	43	40
Posterior cingulate cortex	−1	−32	26	2440				
Inferior temporal gyrus/fusiform area					42	−52	−12	648
Superior temporal gyrus	−50	−28	2	648				
Temporal pole					40	8	−28	648
	−34	18	−22	648				
Caudate nucleus					54	−32	8	648
	−8	20	0	648				
					17	1	23	1024
					10	16	5	1136
Insula					39	−6	−7	2632
	−42	−9	−6	2112				
	−36	18	−4	648				
	−34	−22	10	608				
SMA	−1	10	65	1296				
					4	4	52	648
Thalamus	−8	−9	−2	1288				
					10	−24	10	648
Heschel gyrus					45	−17	8	1280
Cerebellum					10	−44	−7	1168
Cerebellar vermis	−2	−58	−10	648				
Putamen	−22	7	−8	1024				
	−18	19	−9	896				
Precuneus					8	−51	22	976
Parahippocampal region (Amygdala: 10%)					19	2	−13	896
Pallidum					22	−1	6	888
Rolandic operculum					60	−14	12	648
Postcentral gyrus	−62	−4	22	600				
Lingual gyrus (Hippocampus: 40%)	−11	−38	−10	424				

**Table A5:** **Common activation peaks between intention understanding and passionate love studies**.

**Label**	**Left hemisphere**				**Right hemisphere**			
	**MNI coordinates**			**Number of voxels**	**MNI coordinates**			**Number of voxels**
	***x***	***y***	***z***		***x***	***y***	***z***	
dlPFC					2	41	36	32
	−5	54	19	2000				
vmPFC					9	45	−11	280
					4	55	−11	1904
Superior frontal gyrus (BA 6: 40%)					30	−12	62	424
Inferior frontal gyrus (p. Orbitalis)					39	31	−4	824
Inferior frontal gyrus (p. Triangularis) (BA 45: 60%)					50	30	16	632
Inferior frontal gyrus (p. Triangularis) (BA 45: 60%; BA 44: 20%)	−46	24	21	5232				
Inferior frontal gyrus (p. Opercularis)					39	18	13	232
Inferior frontal gyrus (p. Opercularis) (BA 44: 40%)					56	9	21	272
Postcentral gyrus (IPC/PFop: 30%; OP4: 30%; OP3: 10%)					62	−16	23	1248
Postcentral gyrus (BA 2: 80%; SPL/7PC: 20%; BA 3b: 10%)					28	−42	57	1104
SMA (BA 6: 70%)					4	−5	48	48
SMA	−6	21	47	2024				
SMA (BA 6: 60%)	−10	−5	69	80				
Inferior temporal gyrus	−49	−51	−20	3032				
Middle temporal gyrus (V5: 10%)	−53	−68	2	2312				
Superior temporal gyrus (OP1: 10%)	−56	−30	6	2424				
Superior temporal gyrus (IPC/PF: 60%; IPC/PFm: 30%; PGa: 20%)	−60	−46	23	1648				
Supra marginal gyrus (IPC/PFm: 70%; IPC/PF: 30%; IPC/PGa: 30%)					61	−45	24	5800
Anterior cingulate cortex	−7	40	−2	744				
	−7	30	6	224				
					6	34	22	3696
Posterior cingulate cortex					7	−40	14	3024
	−2	−53	29	1088				
Superior parietal lobule (SPL/7PC: 30%; SPL/7A: 20%; hIP3: 10%)	−26	−53	53	1096				
Lingual gyrus (BA 17: 50%; BA 18: 10%)					29	−59	3	904
Inferior temporal gyrus					46	−56	−23	776
Cuneus (BA 18: 10%; BA 17: 10%)					16	−77	18	672
Precuneus					25	−45	8	568
Precuneus (SPL/5L: 40%; SPL/7a: 20%; SPL/5M: 10%)	−14	−50	57	16				
Anterior insula (Id1: 40%)					43	−4	−14	272
Anterior insula					35	28	5	256
Hippocampus					37	−14	−19	1224
Putamen	−27	10	−6	3488				
Fornix	−3	−3	11	3368				
Calcarine gyrus (BA 17: 50%; BA 18: 10%)					14	−87	14	1424
Calcarine gyrus (BA 17: 80%; BA 18: 10%)					21	−95	−2	896
Optic radiations	−26	−36	11	1608				
	−33	−54	−2	56				

*Note: Activations of 10 voxels or above are reported here. All brain areas reported are significant at p < *0.001*, corrected*.

Abbreviations *dlPFC* *dorsolateral Prefrontal Cortex* *vmPFC* *ventro-medial Prefrontal Cortex* *SMA* *Supplementary Motor Area* *STG* *Superior Temporal Gyrus* *IPC/PF* *part of Inferior Parietal Cortex* *IPC/PFm* *part of Inferior Parietal Cortex* *IPC/PGp* *part of Inferior Parietal Cortex* *PGa* *part of Parietal area* *hIP1* *human Intraparietal area 1 (Choi et al., 2006)* *hIP3* *human Intraparietal area 1 (Choi et al., 2006)* *Insula/Ig1 (Insular Lobe granular area; Kurth et al., 2010)* *60%* *TE1.1* *part of the Primary Auditory Cortex (Morosan et al., 2001)* *OP1* *part of Parietal Operculum (Eickhoff et al., 2006)* *OP2* *part of Parietal Operculum (Eickhoff et al., 2006)* *SPL/7PC* *part of Superior Parietal Lobule* *SPL/7A* *part of Superior Parietal Lobule* *SPL/5M* *part of Superior Parietal Lobule (Duvernoy and Bourgoin, 1999)*.
